# Potential of Glutamate-Based Drug Discovery for Next Generation Antidepressants

**DOI:** 10.3390/ph8030590

**Published:** 2015-09-17

**Authors:** Shigeyuki Chaki, Kenichi Fukumoto

**Affiliations:** Pharmacology I, Pharmacology Laboratories, Taisho Pharmaceutical Co., Ltd., 1-403 Yoshino-cho, Kita-ku, Saitama, Saitama 331-9530, Japan; E-mail: k-fukumoto@so.taisho.co.jp

**Keywords:** ketamine, GluN2B antagonist, mGlu2/3 antagonist, mGlu5 antagonist, GLYX-13

## Abstract

Recently, ketamine has been demonstrated to exert rapid-acting antidepressant effects in patients with depression, including those with treatment-resistant depression, and this discovery has been regarded as the most significant advance in drug development for the treatment of depression in over 50 years. To overcome unwanted side effects of ketamine, numerous approaches targeting glutamatergic systems have been vigorously investigated. For example, among agents targeting the NMDA receptor, the efficacies of selective GluN2B receptor antagonists and a low-trapping antagonist, as well as glycine site modulators such as GLYX-13 and sarcosine have been demonstrated clinically. Moreover, agents acting on metabotropic glutamate receptors, such as mGlu2/3 and mGlu5 receptors, have been proposed as useful approaches to mimicking the antidepressant effects of ketamine. Neural and synaptic mechanisms mediated through the antidepressant effects of ketamine have been being delineated, most of which indicate that ketamine improves abnormalities in synaptic transmission and connectivity observed in depressive states via the AMPA receptor and brain-derived neurotrophic factor-dependent mechanisms. Interestingly, some of the above agents may share some neural and synaptic mechanisms with ketamine. These studies should provide important insights for the development of superior pharmacotherapies for depression with more potent and faster onsets of actions.

## 1. Introduction

Recent evidence clearly indicates that targeting glutamatergic transmission is an effective and useful approach to treating depression, as represented by recent ground-breaking clinical findings for the non-competitive N-methyl-d-aspartate (NMDA) receptor antagonist ketamine. To date, ketamine has been demonstrated to be effective not only for patients with depression, including treatment-resistant depression (TRD) [1,2,3,4,5], but also for patients with bipolar depression [6,7], the effects of which became apparent within a few hours and lasted for a week. However, the use of ketamine has some drawbacks, including psychotomimetic and dissociative symptoms after ketamine injection. Also, abuse potential and neurotoxicity following chronic treatment with ketamine should be additional concerns, and ketamine must be injected either intravenously or subcutaneously; both of these matters prevent the routine use of ketamine in daily practice.

To overcome the drawbacks of ketamine, alternatives to ketamine with fewer safety and usage concerns are required; this demand has led to an eruption of research efforts to delineate the molecular and neural mechanisms underlying the rapid and sustained antidepressant effects of ketamine. Based on findings from these research activities, agents acting on both NMDA receptors and metabotropic glutamate (mGlu) receptors have been proposed as alternatives, and the efficacies of some of these agents have been tested in clinical studies, with both encouraging and discouraging outcomes [8,9,10,11,12].

In this review, first, after a brief description of the clinical outcomes of ketamine, the mechanisms underlying the antidepressant effects of ketamine that have been proposed to date will be discussed on both a molecular and a synaptic basis. Then, the potential of possible alternatives, including agents acting on either NMDA receptors or mGlu receptors, will be discussed in the context of clinical evidence as well as their similarities to the efficacy and mechanisms of ketamine. Finally, questions that remain to be solved so as to develop better pharmacotherapies from ketamine research will be raised.

## 2. Clinical and Preclinical Studies of Ketamine

### 2.1. Clinical Studies of Ketamine

The antidepressant effect of ketamine was first demonstrated in 2000 [13], and this effect was confirmed in a subsequent study in which ketamine was found to exert rapid (> 2 h) and sustained (~1 week) effects in patients with TRD following a single injection [5]. To date, several studies have replicated the rapid and sustained antidepressant effects of ketamine [1,2,4], including a two-site, parallel-arm, randomized controlled trial using an active placebo control condition, the anesthetic benzodiazepine midazolam, to optimize blinding and to mitigate the influence of nonspecific factors on antidepressant outcome [3]. In addition to its efficacy for the treatment of patients with TRD, ketamine infusion has been demonstrated to be effective for patients with treatment-resistant bipolar depression [6,7]. The repeated administration of ketamine has also been tested, as first reported by [14], followed by a larger sample of subjects with TRD (including participants from the original study) [15]. In this study, patients with TRD received 6 intravenous infusions of ketamine over a 12-day period, and the overall response rate at the end of the study was 70.8%. The response rate at the end of the study was highly predicted by the response rate at 4 h after the first infusion of ketamine, and among responders, the average time until relapse after the last infusion was 18 days. The efficacy of repeated-dose ketamine was confirmed in a subsequent study, in which ten depressed patients were treated with twice-weekly ketamine infusions until either remission was achieved or four infusions had been administered [16]. In this test, five patients achieved remission, and of these five patients, the improvements in two patients were sustained throughout the 4-week follow-up period.

### 2.2. Mechanisms of Action

The molecular and synaptic mechanisms of the antidepressant effects of ketamine have been actively investigated. To date, several studies have indicated that ketamine’s effects are mediated through its actions on the NMDA receptor, where it acts as an open channel, noncompetitive antagonist [17]. NMDA receptor blockade triggers subsequent events that may explain the rapid and potent antidepressant actions of ketamine [18,19,20]. These studies have revealed enhanced synaptic plasticity/synaptogenesis via several molecular and cellular mechanisms: the synthesis of brain-derived neurotrophic factor (BDNF) and secretion from the dendritic spines, mammalian target of rapamycin (mTOR) activation, and glycogen synthase kinase-3β (GSK3β) inhibition. More specifically, the blockade of NMDA receptors on the GABA interneurons in the medial prefrontal cortex (mPFC) by ketamine disinhibits the pyramidal neurons, leading to increased glutamate release and, in combination with extrasynaptic NMDA receptor (presumably GluN2B-containing NMDA receptor) blockade, increased α-amino-3-hydroxy-5-methyl-4-isoxazolepropionic (AMPA) receptor activity. AMPA receptor activation leads to increases in BDNF and the activation of mTOR signaling, resulting in overall synaptogenesis and synaptic potentiation in the mPFC. Moreover, ketamine has also been reported to increase BDNF translation by inhibiting eukaryotic elongation factor 2 (eEF2) kinase and the subsequent reduction of eEF2 phosphorylation [21]. The involvement of BDNF in the antidepressant effects of ketamine has been confirmed in both rodents and humans. For example, the antidepressant effects of ketamine disappeared in mice in which BDNF signaling had been blocked in some genetic or pharmacological manner [21,22,23]. Moreover, major depressive disorder (MDD) patients with the Val/Val BDNF allele at rs6265 have been reported to be more likely to exhibit an increased antidepressant response to ketamine than Met carriers [24], which is in good agreement with the results obtained in rodents [23]. The actions of ketamine on the mPFC have been underpinned by microinjection and optogenetic studies [25]. The microinjection of ketamine into the infralimbic PFC resulted in acute and long-lasting antidepressant/anxiolytic effects when evaluated using the forced swimming test (FST) and the novelty-suppressed feeding test (NSFT), thereby reproducing the effects observed after the systemic administration of ketamine. Moreover, the optogenetic stimulation of excitatory neurons in the infralimbic PFC not only mimicked these effects, but also increased the number and function of spine synapses. Interestingly, these phenotypes were not reproduced by either the microinjection of ketamine into the prelimbic PFC or optogenetic stimulation of the site.

Although the blockade of NMDA receptors has been hypothesized to play a role in the antidepressant effects of ketamine [26,27], the NMDA receptor subunits and cell type populations in which ketamine acts to trigger rapid synaptic and behavioral responses still remain unclear and controversial. Recently, the blockade of NMDA receptors on fast-spiking GABA interneurons in the mPFC, as described above, has been proposed to mediate the behavioral and synaptic effects of ketamine. However, there are reports that oppose this hypothesis. First, knockout mice lacking GluN1 on parvalbumin-expressing GABA interneurons did not exhibit any impact on depressive-like behavior as evaluated using the FST or the antidepressant effects of ketamine [28], and the antidepressant effect of a selective GluN2B antagonist was intact in knockout mice lacking GluN1 on forebrain interneurons [29]. In contrast, knockout mice lacking GluN2B in pyramidal cortical neurons were reported to exhibit a phenotype resembling that induced by treatment with ketamine: reduced immobility on the FST and TST as well as the increased phosphorylation of mTOR and the synthesis of BDNF and GluA1 [30]. Moreover, the antidepressant and synaptic effects of ketamine were no longer observed in GluN2B knockout mice, suggesting that ketamine may block GluN2B-containing NMDA receptors in pyramidal cortical neurons to exert its antidepressant effects. In addition, knockout mice lacking GluN2A displayed antidepressant-like behavior [29]. However, in these studies, knockout mice showed a robust increase in locomotor activity that may have affected the outcomes of the behavioral studies, and the GluN2B knockout mice did not show any effects in other paradigms, such as sucrose preference or novelty-suppressed feeding. Thus, these data should be interpreted with caution, and further studies are needed to elucidate the cell types responsible for the primary actions of ketamine.

Other mechanisms underlying the antidepressant effects of ketamine have been proposed. Ketamine has been reported to exert anti-inflammatory effects [31]. Consistent with this finding, ketamine has been reported to prevent depressive-like behaviors induced by lipopolysaccharide (LPA) when examined using the FST, coinciding with a reduction in the LPS-induced increases in IL-1β and IL-6 in the PFC [32]. In addition, ketamine has been postulated to prevent the LPS-induced expression of pro-inflammatory cytokines (IL-1β, IL-6, TNF-α) from activated astrocytes by suppressing NF-κB activation through a reduction in the expression of Toll-like receptor 4 [33]. Given that an increase in pro-inflammatory cytokines in the PFC has been proposed to be responsible for the reduced synaptic connectivity observed in depression [34], it is conceivable that the antidepressant effects of ketamine may partly be mediated through the prevention of pro-inflammatory cytokines, although the role of NMDA receptor blockade in this pathway has not been elucidated. In contrast, views differ regarding the effects of ketamine on LPS-induced depressive-like behaviors. The effects of ketamine have been reported to be mediated through the blockade of NMDA receptor activation induced by quinolinic acid (an endogenous NMDA receptor agonist), which is produced as a consequence of the stimulation of the kynurenine pathway by LPS but not through a reduction in IL-1β or IL-6 [35].

Moreover, mechanisms other than NMDA receptor blockade have also been proposed. Ketamine is a racemic mixture containing equal parts of *R*(−)-ketamine and *S*(+)-ketamine. *R*(−)-ketamine reportedly has a lower affinity for the NMDA receptor and a weaker anesthetic potency than *S*(+)-ketamine [36,37]. A recent study has suggested that *R*(−)-ketamine exhibits more potent and longer-lasting antidepressant effects in rodents [38]. Indeed, *R*(−)-ketamine attenuated depressive-like behaviors (increased immobility in the TST and FST and decreased sucrose preference) observed in juvenile mice after neonatal dexamethasone exposure more potently and persistently than *S*(+)-ketamine [38]. This result suggests that ketamine may exert antidepressant effects through mechanisms other than NMDA receptor blockade. Non-NMDA receptor mechanisms have also been suggested by findings concerning the actions of ketamine metabolite’s. Because the plasma half-life of ketamine is approximately 4 min and the overall terminal plasma half-life is 1–3 h [39], the metabolites of ketamine are presumed to play some roles in the sustained antidepressant effects of ketamine. To date, an N-demethylated metabolite, norketamine, has been considered to be an active metabolite of ketamine because it has an anesthetic effect and increases locomotor activity caused by NMDA receptor blockade. However, ketamine is extensively and stereoselectively transformed by multiple hepatic cytochrome P450 isoforms into a broad array of metabolites, including diastereomeric hydroxyketamines, diastereomeric hydroxynorketamines, and (*R*,*S*)-dehydronorketamine [40], and potential associations between the antidepressant response and the plasma concentrations of some of the hydroxynorketamine metabolites have been suggested [41]. Among these metabolites, (2*S*,6*S*)-hydroxynorketamine has received attention because of its pharmacological properties [42]. (2*S*,6*S*)-Hydroxynorketamine has been reported to be a negative allosteric modulator of nicotinic α7 receptor without affinity for the NMDA receptor and to inhibit serine racemase via the inhibition of the α7 receptor. Moreover, when (2*S*,6*S*)-hydroxynorketamine is administered systemically, it enters the brain and stimulates the phosphorylation of mTOR and its downstream targets in the PFC of rats [43]. Therefore, (2*S*,6*S*)-hydroxynorketamine may be an active metabolite involved in the antidepressant effect of ketamine through a mechanism that is independent of direct NMDA receptor blockade.

## 3. Possible Alternatives to Ketamine

### 3.1. Agents Acting on NMDA Receptors

Although mechanisms other than NMDA receptor blockade may be involved in the antidepressant effects of ketamine, as mentioned above, NMDA receptor blockade has been considered to be the major mechanism by which ketamine acts, and some approaches with different effects on the NMDA receptor have been investigated to avoid the adverse effects of ketamine. One approach is the development of subtype-selective NMDA receptor antagonists, with attention focused on the GluN2B subtype. CP-101,606 (traxoprodil), a selective GluN2B antagonist, has been demonstrated to produce an antidepressant effect at a dose with a low potential for producing dissociative effects in patients with TRD (30 patients) after a single intravenous infusion; furthermore, 78% of CP-101,606-treated patients maintained their response status for one week, and 32% maintained their response status for 30 days after the infusion [9]. Subsequently, MK-0657, another selective GluN2B antagonist was tested in a small, randomized, double-blind, placebo-controlled, crossover pilot study, and an oral formulation of MK-0657 showed antidepressant action in patients with TRD (5 patients) as early as day 5, although no improvement was observed when assessed using MADRS as a primary efficacy measure [8]. MK-0657 is currently in Phase 2 clinical development for MDD by Cercor under the name CERC-301. Of note, a recent report has indicated that selective GluN2B antagonists can cause transient cognitive impairments in multiple cognitive domains in non-human primates [44], which should also be taken into account.

A second approach is the development of NMDA receptor channel blockers with lower receptor trapping, since these blockers may be associated with lower rates of associated psychotomimetic effects, compared with ketamine [45]. In a double-blind, randomized, crossover, placebo-controlled study, a single intravenous dose (150 mg) of the low-trapping NMDA antagonist AZD6765 (lanicemine) exerted rapid but short-lived antidepressant effects (effects were only observed at 80 and 110 min after infusion) in 22 patients with TRD without increased psychosis or dissociation [12]. Of note, the antidepressant effect of AZD6765 was not as robust as that of ketamine. In a subsequent larger trial, repeated adjunctive intravenous infusions of AZD6765 at two doses (100 and 150 mg) showed antidepressant effects in patients with TRD at week 2 without psychotomimetic and dissociative side effects, and the effects lasted for 2 weeks after the cessation of infusion [11]. Unfortunately, a recently completed follow-up study exploring the longer-term efficacy of AZD6765 was unable to replicate the clinical efficacy findings [46].

**Figure 1:**
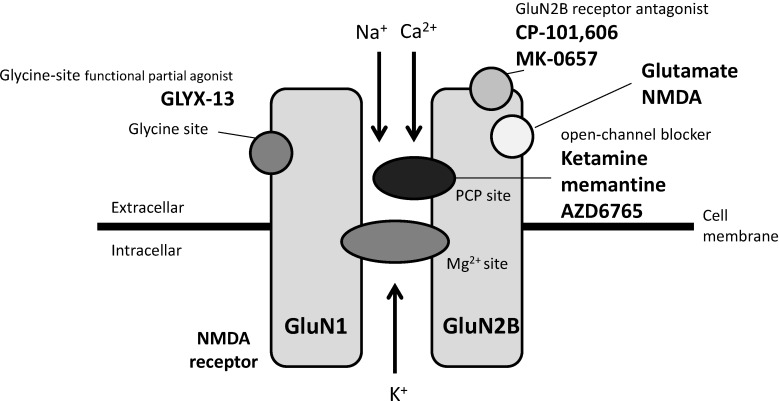
Antidepressant candidates acting on NMDA receptor.

A third approach is the development of agents that act on the glycine modulatory site of the NMDA receptor complex. A single intravenous dose of GLYX-13, a functional partial agonist of the glycine modulatory site [47], has been reported to reduce depressive symptoms within 2 h in patients with TRD in a double-blinded, randomized, placebo-controlled study, and the effects lasted for a week [10]. Moreover, in a 6-week double-blinded trial performed in 40 patients with major depression, sarcosine, an endogenous agonist of the glycine modulatory site, was reported to enable greater improvements in the HAM-D and GAF scores than citalopram throughout the study [48]. Of note, a double-blind, placebo-controlled study of 32 subjects showed that another non-competitive NMDA receptor antagonist, memantine (currently approved by the U.S. Food and Drug Administration for the treatment of Alzheimer disease), administered at doses of 5-20 mg/day in an 8-week trial, was not effective for the treatment of patients with MDD [49]. Interestingly, memantine and ketamine were recently shown to have different NMDA receptor function blocking abilities under physiological concentrations of Mg^2+^, at which ketamine effectively blocks the NMDA receptor while memantine has a negligible effect [50]. Moreover, memantine did not inhibit eEF2 phosphorylation nor did it increase BDNF expression under the above-mentioned condition. The different abilities of memantine and ketamine to block NMDA receptor function may provide a clue regarding the dissociation between the potent antidepressant effects and the adverse effects of ketamine. Agents acting on NMDA receptor described above are summarized in Figure 1.

In addition to focusing on agents acting on the NMDA receptor in different ways from that of ketamine, combination with lithium has been proposed as a possible means of reducing unwanted side effects of ketamine. Lithium combination has been reported to reduce the effective dose of ketamine, which is accompanied by synaptic changes in the mPFC, presumably through the inhibition of GSK3β [51,52]. Moreover, lithium combination prolonged both the antidepressant effects and the restoration of dendritic spine density in the mPFC of stressed mice treated with ketamine for at least 2 weeks, presumably by reducing ketamine-induced oxidative stress [51].

### 3.2. Agents Acting on mGlu Receptors

While NMDA receptor has critical roles in facilitating fast synaptic transmission, mGlu receptors, other glutamate receptor subtypes which are coupled to G-proteins, have critical roles as modulators of synaptic transmission. In addition to targeting the NMDA receptor, the manipulation of mGlu receptors has gained attention as an alternative to ketamine for the treatment of depression. Among these manipulations, both mGlu2/3 receptor blockade and mGlu5 receptor blockade have been proposed as useful approaches. mGlu2/3 receptor antagonists, such as MGS0039 and LY341495, have been reported to exert antidepressant effects in various animal models [53,54,55,56]. In addition, an mGlu2/3 receptor antagonist has been reported to exert rapid and sustained antidepressant effects in a chronic unpredictable stress model and to induce an antidepressant effect in an animal model refractory to current medications [57,58], both of which resemble the effects of ketamine. Consistent with antidepressant effects in animal models, the mGlu2/3 receptor antagonist has been shown to share some neural and synaptic mechanisms with ketamine. mGlu2/3 receptor antagonists increase glutamate release by blocking presynaptic mGlu2/3 receptor of glutamatergic terminals. Increase in glutamate release leads to postsynaptic AMPA receptor stimulation and the subsequent activation of the tropomyosin-related kinase B receptor and mTOR signaling, cellular events that lead to increased levels of synaptic proteins in the PFC [22,55,59,60,61]. Therefore, these findings suggest that similar to ketamine, the mGlu2/3 receptor antagonist may have the potential to exert rapid and sustained antidepressant effects in patients with TRD. Recently, we reported that serotonergic systems may be involved in the effects of the mGlu2/3 receptor antagonist and of ketamine in the NSFT and that AMPA receptor-dependent 5-HT release and the subsequent stimulation of postsynaptic 5-HT_1A_ receptors may be involved [62], although there are inconsistent findings that the antidepressant effects of ketamine and the mGlu2/3 receptor antagonist were independent of serotonergic systems when evaluated using the FST and the TST, respectively [55,63]. Thus, further investigations are required to elucidate the neural mechanisms of the mGlu2/3 receptor antagonist and ketamine. In a phase I trial, BCI-838, an mGlu2/3 receptor antagonist, did not induce psychotomimetic symptoms, suggesting that the mGlu2/3 receptor antagonist may have a better safety profile than ketamine [64]. Therefore, the mGlu2/3 receptor antagonist may be an attractive alternative to ketamine for the treatment of depression.

mGlu5 receptor antagonists, such as MPEP, MTEP, and GRN-529, reportedly exerted antidepressant effects in several animal models of depression [65,66,67,68], and these effects were mimicked by the behavioral phenotypes of knockout mice lacking the mGlu5 receptor [69]. Regarding the neural mechanisms of the antidepressant effects of mGlu5 receptor antagonists, NMDA receptor blockade and the activation of BDNF signaling have been reported to be involved in these mechanisms (for a review, see Ref. [70]). In addition, the antidepressant effect of MTEP was blocked by pretreatment with an inhibitor of 5-HT synthesis [71], which was confirmed by our recent report that the effect of MPEP is blocked by an inhibitor of 5-HT synthesis in the NSFT [72]. Thus, similar to ketamine and mGlu2/3 receptor antagonists, serotonergic transmission may be involved in the actions of mGlu5 receptor antagonists as well. In contrast, the effects of mGlu5 receptor antagonists were blocked by a 5-HT_2A/2C_ receptor antagonist, but not by a 5-HT_1A_ receptor antagonist, when evaluated using the TST [71] and the NSFT [72], and these effects are different from those produced by the mechanisms of ketamine and mGlu2/3 receptor antagonists. Recently, the mGlu5 receptor antagonist Basimglurant was administered as an adjunctive treatment to ongoing SSRI or SNRI treatment in a randomized, double-blind, placebo-controlled study, and an antidepressant effect was observed in patients with MDD, although the effect did not reach statistical significance when assessed using MADRS as a primary endpoint [73]. Therefore, this mGlu5 receptor antagonist may have a weaker antidepressant effect than ketamine, possibly because its mechanism of mGlu5 receptor antagonism differs from the mechanisms of ketamine. Further clinical studies are needed to examine the usefulness of mGlu5 receptor antagonists as antidepressant drugs.

In addition to the mGlu2/3 receptor and the mGlu5 receptor, the roles of both the mGlu4 receptor and the mGlu7 receptor have also been investigated. Recently, LSP4-2022, an mGlu4 receptor agonist, has been reported to induce a pro-depressant effect in wild-type mice but not in knockout mice lacking the mGlu4 receptor when evaluated using the TST, suggesting that the inhibition of the mGlu4 receptor may induce antidepressant effects [74]. In contrast, a contradictory result was also obtained, as the mGlu4 receptor positive allosteric modulator (PAM) known as ADX88178 was found to exert an antidepressant effect when evaluated using the FST [75]. Moreover, previous reports have indicated that an mGlu4 receptor agonist and PAM did not induce antidepressant effects [76,77]. Thus, further study is needed using the mGlu4 receptor antagonist to elucidate the roles of the mGlu4 receptor in depression. The mGlu7 receptor agonist (agoPAM) AMN082 has been reported to exert antidepressant effects when evaluated using the FST and TST [78,79], and these effects were not observed in knockout mice lacking the mGlu7 receptor [80] or after co-administration with an mGlu7 receptor antagonist, suggesting that the mGlu7 receptor stimulation may play an important role in antidepressant effects [81]. Also, recently, the antidepressant effect of AMN082 has been reported to be mediated through mTOR signaling, leading to an increase in the levels of synaptic proteins in the PFC, as observed for ketamine, while unlike ketamine, its effect was not mediated through AMPA receptor activation and was no longer present at 23 h after treatment [82]. In contrast, mGlu7 receptor blockade has been suggested to show antidepressant/anxiolytic effects [83]. Thus, further investigation of the antidepressant effects of mGlu7 receptor agonists and comparisons of their neural mechanisms with those of ketamine may be warranted to investigate the clinical efficacy of mGlu7 receptor agonists for the treatment of depression.

In addition to agents acting on mGlu receptors, it should be mentioned that scopolamine has been reported to exert rapid and sustained antidepressant effects in both rodents and humans, and scopolamine may share some of synaptic and neural mechanisms with ketamine, including glutamate surge in the mPFC [84,85].

## 4. Conclusion and Future Directions

**Figure 2:**
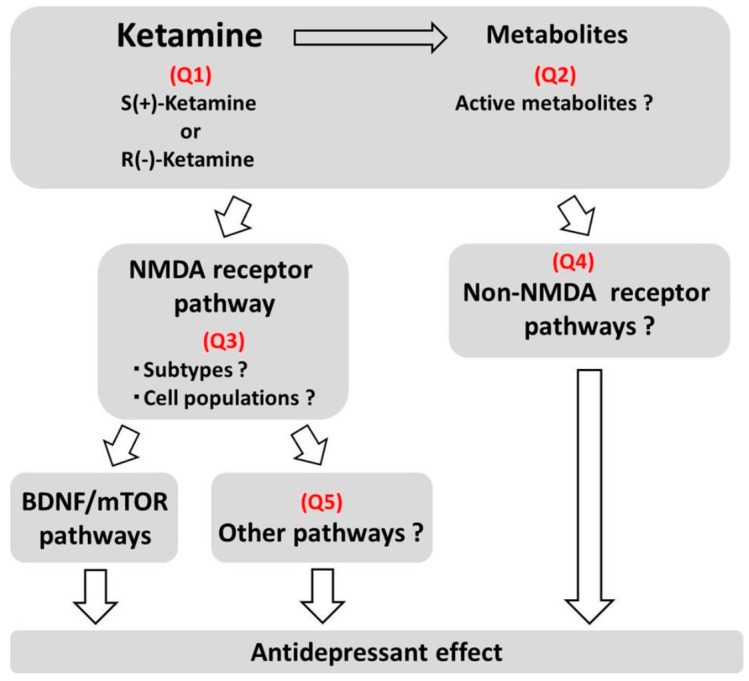
Issues to be solved on mechanisms of antidepressant effects of ketamine.

As discussed above, elucidating the mechanisms underlying the antidepressant effects of ketamine is an important step towards developing more effective and safer pharmacotherapies for the treatment of depression. For this purpose, the following issues, described in Figure 2, should be resolved.

Q1. Which stereoisomer is more active for the treatment of depression?

Of the ketamine stereoisomers, *S*(+)-ketamine has been considered to be the more active isomer, since *S*(+)-ketamine has a higher NMDA receptor blockade activity and a more potent anesthetic activity than *R*(−)-ketamine. However, recent animal studies have suggested that *R*(−)-ketamine has a more potent and sustained antidepressant effect than *S*(+)-ketamine. Therefore, the active isomer producing the antidepressant effect should be reconsidered and investigated in other animal models and possibly in patients with depression.

Q2. Which metabolites are involved in the antidepressant effects of ketamine?

Ketamine is extensively and stereoselectively transformed into a broad array of metabolites, and these metabolites, which have been previously considered to be inactive metabolites, may have roles in the antidepressant effects of ketamine. Thus, the roles of these metabolites and their mechanisms should be elucidated.

Q3. Which NMDA receptor subtype is involved and which cell populations express the NMDA receptor that is involved in the antidepressant effects of ketamine?

Although it is presumed that the blockade of NMDA receptors on GABA interneurons may trigger the induction of a glutamate surge, leading to subsequent events that enhance synaptic connectivity in the mPFC, this hypothesis needs to be proven. Moreover, although selective GluN2B antagonists exert rapid and sustained antidepressant effects as well as increase the synaptic connections, clinical evidence for GluN2B antagonists has not been as promising as expected. Thus, the subunits comprising the NMDA receptor should be reconsidered, and the mode of action responsible for receptor blockade should be investigated.

Q4. What are the potential roles of non-NMDA receptor pathways?

Although NMDA receptor blockade has been proposed as the main mechanism responsible for the antidepressant effects of ketamine, the possible roles of mechanisms acting independent of the NMDA receptor or resulting in the indirect blockade of the NMDA receptor should be investigated, as exemplified by the effects of stereoisomers and metabolites of ketamine.

Q5. Are pathways other than the BDNF-mTOR pathway involved in the antidepressant effects of ketamine?

Although the roles of BDNF (which may stimulate the mTOR pathway) have been suggested, the roles of other pathways independent of the BDNF-mTOR pathway have been reported, and these pathways should be investigated in connection with synaptic plasticity.

As described above, it is premature to conclude that any single mechanism is solely responsible for the antidepressant response, and the response may be mediated through complex pathways downstream of ketamine’s direct actions at any receptor. The elucidation of the above issues, and eventually a detailed explanation of the neural and synaptic mechanisms of the antidepressant effects of ketamine, should provide useful information for developing next-generation antidepressants that are more effective and are associated with fewer adverse effects. Moreover, studies on agents targeting mGlu receptors as possible alternatives to ketamine have just started, and these possibilities should be more intensively investigated both in preclinical and clinical studies.
